# Increasing Ca^2+^ in photoreceptor mitochondria alters metabolites, accelerates photoresponse recovery, and reveals adaptations to mitochondrial stress

**DOI:** 10.1038/s41418-019-0398-2

**Published:** 2019-08-02

**Authors:** Rachel A. Hutto, Celia M. Bisbach, Fatima Abbas, Daniel C. Brock, Whitney M. Cleghorn, Edward D. Parker, Benjamin H. Bauer, William Ge, Frans Vinberg, James B. Hurley, Susan E. Brockerhoff

**Affiliations:** 1 Biochemistry Department, University of Washington, Seattle, WA 98109 USA; 2 John A. Moran Eye Center, University of Utah, Salt Lake City, UT 84132 USA; 3 Opthalmology Department, University of Washington, Seattle, WA 98109 USA

**Keywords:** Calcium channels, Metabolomics, Cell biology, Neuroscience

## Abstract

Photoreceptors are specialized neurons that rely on Ca^2+^ to regulate phototransduction and neurotransmission. Photoreceptor dysfunction and degeneration occur when intracellular Ca^2+^ homeostasis is disrupted. Ca^2+^ homeostasis is maintained partly by mitochondrial Ca^2+^ uptake through the mitochondrial Ca^2+^ uniporter (MCU), which can influence cytosolic Ca^2+^ signals, stimulate energy production, and trigger apoptosis. Here we discovered that zebrafish cone photoreceptors express unusually low levels of MCU. We expected that this would be important to prevent mitochondrial Ca^2+^ overload and consequent cone degeneration. To test this hypothesis, we generated a cone-specific model of MCU overexpression. Surprisingly, we found that cones tolerate MCU overexpression, surviving elevated mitochondrial Ca^2+^ and disruptions to mitochondrial ultrastructure until late adulthood. We exploited the survival of MCU overexpressing cones to additionally demonstrate that mitochondrial Ca^2+^ uptake alters the distributions of citric acid cycle intermediates and accelerates recovery kinetics of the cone response to light. Cones adapt to mitochondrial Ca^2+^ stress by decreasing MICU3, an enhancer of MCU-mediated Ca^2+^ uptake, and selectively transporting damaged mitochondria away from the ellipsoid toward the synapse. Our findings demonstrate how mitochondrial Ca^2+^ can influence physiological and metabolic processes in cones and highlight the remarkable ability of cone photoreceptors to adapt to mitochondrial stress.

## Introduction

Photoreceptors, the neurons that initiate vision, must survive in a hostile cellular environment. In the retina they are exposed to damaging light radiation, experience 100-fold fluctuations in intracellular Ca^2+^, are located near blood vessels with high levels of O_2_, and use ATP faster than most other types of cells. Despite these chronic stressors, most people retain vision throughout their lives, highlighting the extraordinary ability of photoreceptors to regulate cellular homeostasis and maintain viability.

Maintenance of Ca^2+^ homeostasis is critical for photoreceptor function and survival. Photoreceptors rely on Ca^2+^ as a second messenger for recovery from transient light signals, adaptation to constant illumination, and neurotransmission [1, 2]. Both chronic elevations and chronic decreases in cytosolic Ca^2+^ have been implicated in photoreceptor cell death and retinal disease [3, 4]. Ca^2+^-associated cell death is often mediated by mitochondria, as mitochondrial Ca^2+^ overload triggers opening of the mitochondrial permeability transition pore (mPTP) and subsequent cell death [5]. Accordingly, increases in cellular Ca^2+^ in isolated rat retinas cause photoreceptor-selective apoptosis that depends on mPTP activity [6].

Mitochondrial Ca^2+^ uptake can also be beneficial to the cell. Cytosolic Ca^2+^ is buffered by mitochondria, and in photoreceptors the precise localization of mitochondria to the ellipsoid can protect the cell body from the cytosolic Ca^2+^ that accumulates in the outer segment in darkness [7–9]. Uptake of Ca^2+^ into mitochondria can also influence their energetic output [10]. However, metabolic responses to changes in mitochondrial Ca^2+^ vary across tissues, reflecting the diverse metabolic demands of different cell types [11].

Ca^2+^ import into the mitochondrial matrix occurs via the mitochondrial Ca^2+^ uniporter complex (MCU), comprised of a multimer of the pore-forming protein MCU and many associated regulatory proteins [12–14]. The protein EMRE is necessary for MCU function in vertebrates, while MICU proteins (MICU1-3) tune Ca^2+^ uptake through the uniporter complex [15–18]. This degree of regulation, along with the variability of modulator expression across tissues, implies that the activity of the MCU complex is attuned to cellular needs and critical in the interplay between optimal function and prolonged survival.

To investigate the relationship between mitochondrial Ca^2+^ and photoreceptor physiology we analyzed expression of MCU and its regulators MICU1, MICU2, and MICU3 in zebrafish retina. While regulator expression is similar between retinal and brain tissue, MCU expression is unusually low in cone photoreceptors. We hypothesized that limiting mitochondrial Ca^2+^ influx via MCU may serve a protective function, so we generated zebrafish models of cone-specific MCU overexpression to test how cones respond to the increase of this key modulator of mitochondrial Ca^2+^.

## Results

### Cones express low levels of MCU

**Fig. 1:**
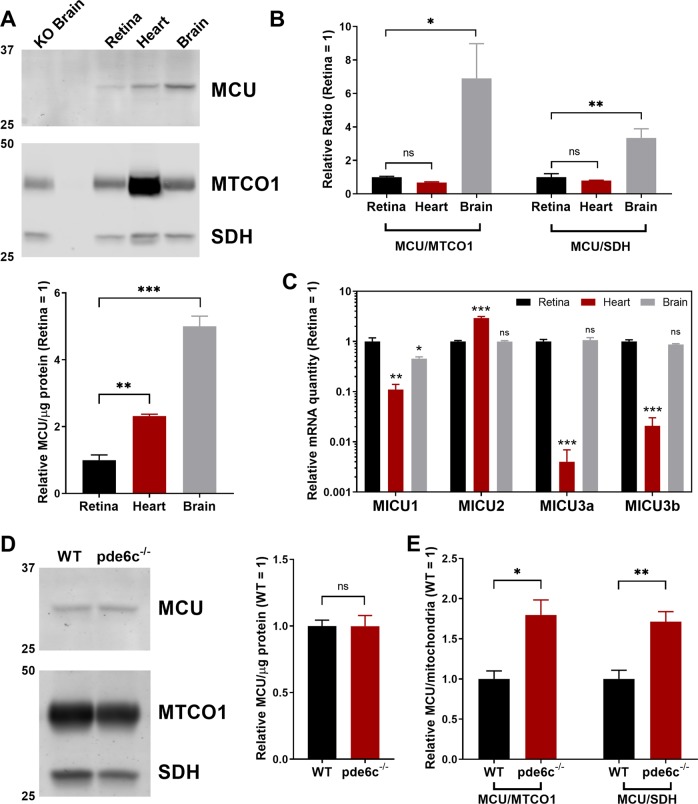
MCU expression is limited in the retina, particularly in cones. **a** Western blot of zebrafish tissue lysate, enriched for mitochondrial proteins and probed for MCU, mitochondrial cytochrome oxidase (MTCO1), and succinate dehydrogenase (SDH). Samples were pooled from either four retinas, two hearts, or one brain and performed with *n* = 3 replicates of each pool. Each lane contains 8 µg of protein. The lower panel shows quantification of the replicates, normalized to retina tissue. The mean is reported and bars = standard error. **p < 0.01, ***p < 0.001 using ANOVA followed by Dunnett post hoc test (comparison with retina). **b** Within each lane from gels analyzed in panel A, the ratios of MCU signal to the mitochondrial proteins MTCO1 and SDH were determined. Values were normalized relative to retina tissue. The mean is reported and bars = standard error. *p < 0.05, **p < 0.01 using ANOVA followed by Dunnett post hoc test (comparison with retina). **c** qRT-PCR quantification of relative mRNA of MICU proteins (relative to reference gene *Ef1α* and/or *b2m*, see “Methods”) across retina, heart, and brain tissues. The mean is reported and bars = standard error. *p < 0.05, **p < 0.01, ***p < 0.001 and ns = not significant using ANOVA followed by Dunnett post hoc test (comparison with retina). **d** Mitochondria-enriched retinal lysate of WT and pde6c^−/−^ cone deficient retinas. Each lane is from lysate of two pooled retinas from a single fish. The right panel shows quantification of replicates; *n* = 4 fish. Each lane contains 30 µg of protein. The mean is reported and bars = standard error. Ns = not significant using Welch’s *t*-test. **e** Relative quantification of the ratio of MCU to mitochondrial proteins MTCO1 and SDH in WT and pde6c^−/−^ cone deficient retinas from experiments shown in D. The mean is reported and bars = standard error. *p < 0.05, **p < 0.01 using Welch’s *t*-test

We developed a custom antibody against amino acids 21-202 of zebrafish MCU and validated its specificity with a global zebrafish MCU knockout (KO, Fig. 1a). MCU protein expression is high in brain, lower in the heart, and lowest in the retina (Fig. 1a). Retina resembles heart when MCU is normalized to mitochondrial proteins cytochrome oxidase (MTCO1) and succinate dehydrogenase (SDH) (Fig. 1b). However, transcript expression of the MCU regulators MICU1, MICU2, and MICU3 resembles brain more than heart (Fig. 1c).

Our antibody was not suitable for immunohistochemistry of endogenous MCU, so we used the pde6c^−/−^ zebrafish model of cone-specific degeneration to estimate MCU expression in cones. In this model, cones degenerate and rod photoreceptors populate the retina [19]. Cones have more mitochondrial volume than rods [20, 21]. Without cones, there are fewer mitochondrial membrane proteins but no significant loss of MCU signal (Fig. 1d, e). This shows that cone mitochondria must have less MCU than mitochondria of other retinal neurons.

### Overexpression of MCU in cones raises basal [Ca^2+^] in the mitochondrial matrix

**Fig. 2:**
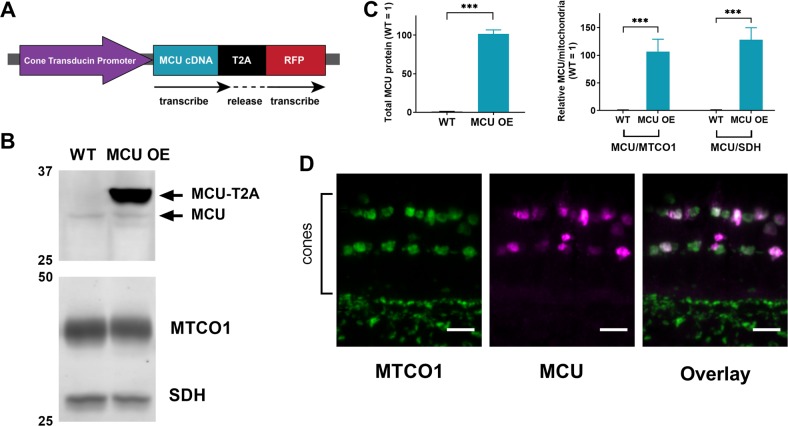
Successful generation of a cone-specific MCU overexpression zebrafish model. **a** Schematic of the MCU OE construct. The cone transducin promoter (TαCP, *gnat2*) drives expression of zebrafish MCU cDNA in all cone subtypes. The MCU cDNA is tagged with a T2A sequence followed by RFP. The T2A sequence causes ribosomes to stall and release the nascent MCU polypeptide with some added peptides from the T2A sequence before translating the RFP separately. Thus, RFP is present in the cytosol of cones with MCU overexpression. **b** Mitochondria-enriched retinal lysate of WT and MCU OE retinas probed with antibodies for MCU, MTCO1, and SDH. Each lane contains 8 µg of protein from lysate of two pooled retinas from a single fish. **c** Quantification of relative MCU signal as a function of protein concentration and relative to other mitochondrial markers from the type of analysis shown in B (*n* = 4 fish). Both exogenous and endogenous MCU were used for total MCU quantification in the MCU OE retina. The mean is reported and bars = standard error. ***p < 0.001 using Welch’s *t*-test. **d** Immunohistochemistry of a larval zebrafish retina expressing the MCU construct in A using MCU and mitochondrial cytochrome oxidase (MTCO1) antibodies. Scale bar = 5 µm

We hypothesized that low expression of MCU in cones could be protective and sought to identify the consequences of increasing mitochondrial Ca^2+^ content. We established a stable transgenic line that uses MCU-T2A-RFP under control of the promoter for cone transducin (“*gnat2*” or “TαCP”) to overexpress zebrafish MCU in cones (Fig. 2a). Because of the T2A sequence, cones overexpressing MCU (MCU OE) also express cytosolic RFP [22]. MCU expression in MCU OE retinas is 102 ± 5-fold higher than normal (Fig. 2b, c). The overexpressed MCU localizes to cone mitochondria (Fig. 2d).

**Fig. 3:**
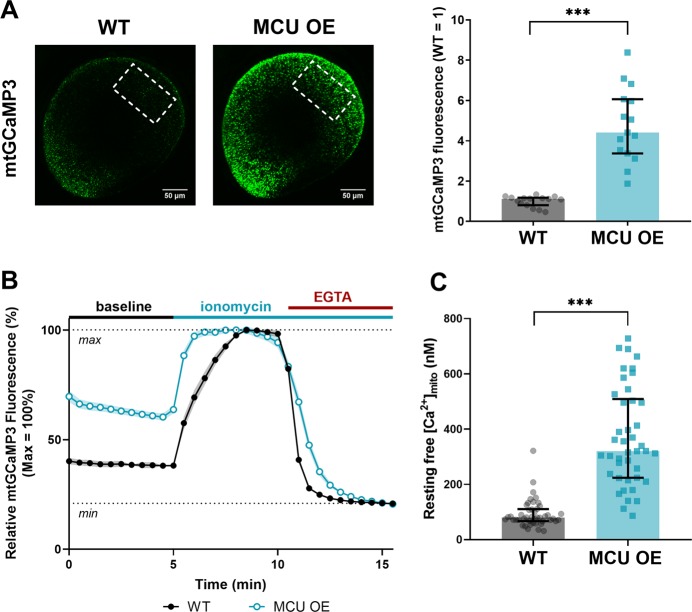
Overexpression of MCU in cones raises basal [Ca^2+^] in the mitochondrial matrix. **a** Total cone mitochondria clusters in a larval zebrafish eye expressing *gnat2*:mito-GCaMP3, a mitochondrial Ca^2+^ sensor (green). Dotted outlines demarcate the region of the eye used for fluorescence quantification. The median is reported with bars = interquartile range, *n* = 15 larvae for both WT and MCU OE. ***p < 0.001 using Mann–Whitney test. **b** Relative mito-GCaMP3 fluorescence of cone mitochondrial clusters in adult retinal slices of *gnat2:*mito-GCaMP3 fish (WT or MCU OE) collected between 3 and 4 months of age. Baseline fluorescence was first assayed in the presence of KRB buffer containing 2 mM CaCl_2_, then ionomycin (5 µM) was added to the slice to allow 2 mM Ca^2+^ entry into the mitochondria to saturate the probe. Next, EGTA (5 mM) was added to the solution (keeping [ionomycin] constant) to chelate Ca^2+^ and establish the minimum GCaMP3 fluorescence signal. *n* = 45 mitochondrial clusters (three fish) for WT and *n* = 42 mitochondrial clusters (three fish) for MCU OE. Slices were imaged every 30 s. The mean is reported and shaded region = standard error. **c** Approximation of resting free [Ca^2+^] in mitochondria clusters assayed in B using the equation $\left[ {Ca^{2 + }} \right] = K_D \times \frac{\theta }{{1 - \theta }}$, where $\theta = \frac{{F_0 - F_{min}}}{{F_{max} - F_{min}}}$ . We used the previously reported *K*_D_ of GCaMP3 (345 nM, from ref. 23) as an approximation for our calculation. Reporting the median with bars = interquartile range and ***p < 0.001 using Mann–Whitney test

Ca^2+^ influx depends on MCU and its regulators, so we investigated whether MCU overexpression alone increases the steady-state concentration of free Ca^2+^ in cone mitochondria. We used *gnat2*:mito-GCaMP3 fish, which express the Ca^2+^ sensor GCaMP3 in cone mitochondria [9]. Mito-GCaMP3 fluorescence in the mitochondrial clusters of live zebrafish larvae is 4.4-fold higher (median, Q1:3.4, Q3:6.06-fold) in MCU OE compared with wild-type (WT) siblings (Fig. 3a). We next prepared ex vivo retinal slices of adult *gnat2*:mito-GCaMP3 zebrafish, measuring the baseline mito-GCaMP3 fluorescence (*F*_0_), the maximum fluorescence (*F*_max_) by addition of ionomycin to the media containing 2 mM Ca^2+^, and the minimum fluorescence (*F*_min_) by addition of 5 mM EGTA to chelate Ca^2+^ (Fig. 3b). Comparing (F_0_−F_min_) with (F_max_−F_min_) indicated that baseline GCaMP3 is at 20 ± 1% of maximum fluorescence in WT mitochondria and 48 ± 2% of maximum in MCU OE mitochondria. Using these measurements and a *K*_D_ of 345 nM for the blinding of Ca^2+^ to GCaMP3 [23], the baseline free [Ca^2+^]_mito_ in WT mitochondria is 80.0 nM (median, with Q1:67.1, Q3:110.5 nM) and in MCU OE mitochondria is 320.6 nM (median, with Q1:223.9, Q3:509.0 nM) (Fig. 3c, equation in legend).

### Cones overexpressing MCU survive through early adulthood despite changes to mitochondrial morphology

**Fig. 4:**
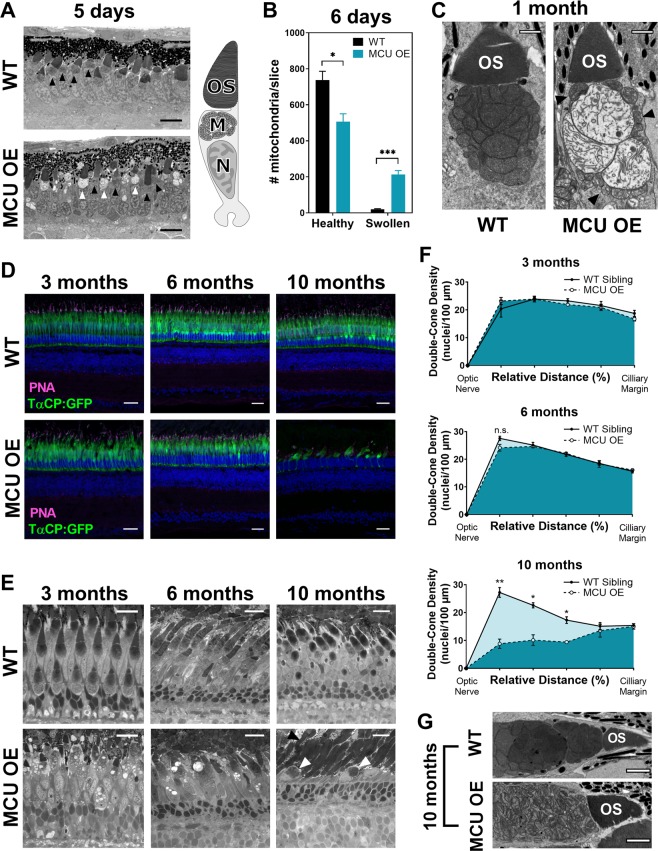
Cones overexpressing MCU survive through early adulthood despite mitochondrial abnormalities, but eventually degenerate. **a** Electron micrograph (EM) of MCU OE cones at 120 h (5 days) of age. MCU OE cone mitochondria are very heterogenous; some are healthy with dense cristae (black arrow) and others are swollen with little cristae density (white arrow). Scale bar = 5 µm. Right: a schematic of a cone cell with the outer segment (OS), mitochondria (M) in the ellipsoid region, and nucleus (N) labelled. **b** Quantification of cone mitochondrial phenotypes from EM images of whole zebrafish larval eyes (single slice at optic nerve) at 6 days of age. *n* = 3 larvae for both WT and MCU OE fish. The mean is reported and bars = standard error. *p < 0.05, ***p < 0.001 using a *t*-test with the Holm–Sidak correction for multiple tests. **c** EM images of cone mitochondria in WT sibling and MCU OE fish at 1 month of age. Mitochondria remain heterogenous in MCU OE cones as they age, with cones containing a mix of healthy mitochondria (black arrows) and swollen mitochondria. Scale bar = 1 µm. **d** WT sibling and MCU OE retinas stained with Hoechst (blue) and exhibiting fluorescence from *gnat2*:GFP in all cone types (green) at 3, 6, and 10 months of age. Cone outer segments are labelled with α-PNA (magenta). Scale bar = 25 µm. **e** WT sibling and MCU OE retinas stained with Richardson’s stain at 3, 6, and 10 months of age. Swollen mitochondria are still observed in 3 and 6 month cones. By 10 months, the cones are very few and have severe morphological disturbances (white arrow); however, rod mitochondria and outer segments remain intact (black arrow). Scale bar = 25 µm. **f** Quantification of double-cone nuclei from the optic nerve to the ciliary margin in WT and MCU OE fish. Counts are an average of dorsal and ventral retinal slices from each eye in each fish, *n* = 3 WT and 3 MCU OE fish. Bars = standard error. *p < 0.05, **p < 0.01 using a *t*-test with Holm–Sidak correction for multiple tests. **g** EM of cone mitochondria from 10-month-old WT and MCU OE fish. The remaining MCU OE cones at 10 months have severe mitochondrial fragmentation. Scale bar = 2 µm

Mitochondria respond to excessive matrix Ca^2+^ by swelling, losing optical/electron density, and potentially opening the mPTP that can trigger cell death [24–26]. Abnormal mitochondria are also observed in animal models of elevated [Ca^2+^]_mito_ [27, 28]. By just 120 h of age, MCU OE cones contain many large, swollen mitochondria that have lost significant cristae and electron density, but otherwise appear normal and survive (Fig. 4a, b, Supplementary Fig. 4A). As MCU OE fish age, cones maintain a mix of both healthy and swollen mitochondria (Fig. 4c, Supplementary Fig. 4B). This heterogeneity is consistent with reports of differential swelling and permeability across a population of mitochondria in response to Ca^2+^ [24, 29, 30].

We next assessed long-term survival of MCU OE cones. By visualizing cones with *gnat2*:GFP expression (Fig. 4d, Supplementary Fig. 4C), analyzing histology with Richardson’s stain (Fig. 4e), and quantifying double-cone nuclei (Fig. 4f) we found that MCU OE cones are preserved at 3 and 6 months of age. Throughout this period, cones maintain a heterogenous population of normal and swollen mitochondria. By 10 months severe cone loss occurs. The remaining cones in 10 month MCU OE retinas no longer have large swollen mitochondria but instead have severely fragmented mitochondria (Fig. 4g), which are associated with apoptosis [31–33]. Rods remain intact and abundant even after cone loss (Supplementary Fig. 4D).

### Retinas with MCU overexpressing cones have increased isocitrate dehydrogenase and α-ketoglutarate dehydrogenase activity

The remarkable survival of MCU OE cones allowed us to determine how altered mitochondrial [Ca^2+^] and structure affect cone metabolism prior to degeneration. Zebrafish retinas are cone dominant; we found cones comprise ~40% of the mitochondrial mass in a zebrafish retina (Fig. 1d, MTCO1 and SDH in cone-deficient retinas are present at 56 ± 4% and 58 ± 5% of WT levels, *n* = 4). Thus, analysis of whole retinas provides valuable information regarding changes to mitochondrial metabolism in cones.

**Fig. 5:**
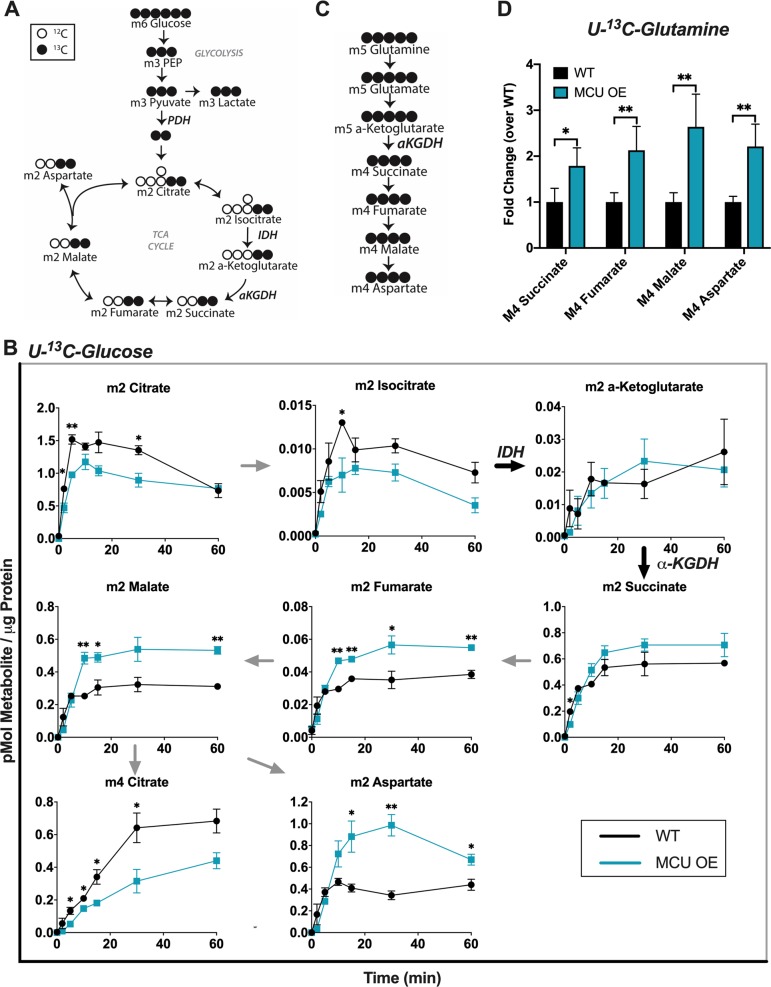
MCU OE cones have increased IDH and α-KGDH activity. **a** Diagram showing how labelled carbons from U-^13^C-glucose are incorporated through glycolysis and the first round of the TCA cycle. Shaded = labeled carbon, empty = unlabeled carbon. **b** Levels of isotopomers in WT and MCU OE retinas supplied with U-^13^C-glucose. “m” signifies the number of ^13^C-labeled carbons in each metabolite. “m2” TCA cycle metabolites are made from one round of the TCA cycle. Data points represent averages from *n* = 3 retinas from three different fish. Fish were 4 months of age. *p < 0.05, **p < 0.01 using Welch’s *t*-test. Bars = standard error. **c** Diagram showing how labelled carbons from U-^13^C-glutamine are incorporated into α-ketoglutarate and downstream metabolites. Shaded = labeled carbon, empty = unlabeled carbon. **d** Levels of isotopomers in WT and MCU retinas supplied with 2 mM ^13^C-glutamine for 15 min. Data points represent averages from *n* = 3 retinas from three different fish. Fish were 4 months of age. *p < 0.05, **p < 0.01 using Welch’s *t*-test. Bars = standard error

In vitro studies have shown that Ca^2+^ lowers the *K*_m_ of isocitrate dehydrogenase (IDH) and α-ketoglutarate dehydrogenase (α-KGDH) for their substrates [34, 35]. We sought to determine if MCU OE in cones alters the activities of these enzymes. Cones rely on glucose as a fuel, so we incubated 4-month-old WT and MCU OE retinas in U-^13^C-glucose and used gas chromatography–mass spectrometry (GC–MS) to quantify ^13^C-labeled TCA cycle metabolites (Fig. 5a).

Steady-state levels of m2 citrate and m2 isocitrate are lower in MCU OE retinas (Fig. 5b). The m2 α-ketoglutarate levels are unchanged, while m2 succinate, m2 fumarate, m2 malate, and m2 aspartate are higher in MCU OE retinas. This is consistent with both IDH and α-KGDH being stimulated by Ca^2+^. Ca^2+^ stimulation of IDH lowers its *K*_m_ for isocitrate, meaning it is more active at lower concentrations and able to deplete isocitrate pools. Increased IDH activity in isolation would cause m2 α-ketoglutarate accumulation, but instead we observe similar steady-state levels of m2 α-ketoglutarate in MCU OE and WT retinas and accumulation of metabolites only downstream of α-KGDH. This suggests that MCU OE retinas also have enhanced α-KGDH activity, preventing the buildup of α-ketoglutarate and increasing production of downstream metabolites. This shift in steady-state levels is also reflected in the total (sum of unlabeled and all isotopomers) metabolite levels (Supplementary Fig. 5G). Additional metabolite data showing unaltered glycolytic activity in MCU OE retinas, full isotopomer distribution at the 30-minute time point, and isotopic enrichment is included in the supplemental material (Supplementary Fig. 5A, B, D, E).

Glucose is a physiologically relevant fuel for photoreceptors, but it does not allow for IDH and α-KGDH activity to be observed in isolation because the two are intrinsically linked in the TCA cycle. To confirm that α-KGDH is stimulated by Ca^2+^ in MCU OE cones, we bypassed IDH and fueled α-KGDH directly with U-^13^C-glutamine (Fig. 5c). MCU OE retinas fueled with U-^13^C-glutamine accumulate higher levels of metabolites downstream of α-KGDH (Fig. 5d, glutamine titration in Supplementary Fig. 5F). These observations confirm that Ca^2+^ enhances α-KGDH activity in zebrafish cones.

It has been reported both in vitro and in vivo that Ca^2+^ can boost pyruvate dehydrogenase (PDH) activity by stimulating PDP1c, a subunit of the phosphatase that converts inactive phosphorylated PDH to active unphosphorylated PDH [35, 36]. We hypothesized MCU OE cones would have a lower ratio of phosphorylated/total PDH due to increased mitochondrial Ca^2+^, but instead it is slightly increased (1.12 ± 0.04-fold higher in MCU OE, *p* < 0.05 using Welch’s *t*-test) (Supplementary Fig. 5C).

### Overexpression of MCU in cones reduces cytosolic Ca^2+^ transients and alters their phototransduction kinetics

**Fig. 6:**
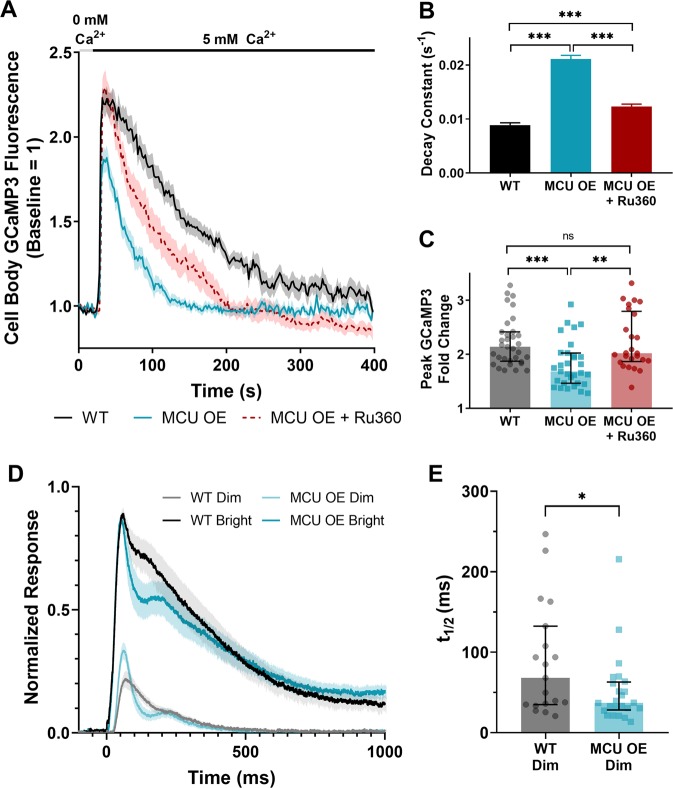
Overexpression of MCU in cones reduces cytosolic Ca^2+^ transients and alters their phototransduction kinetics. **a** Isolated retinas from *gnat2*:GCaMP3 fish preincubated in 0 mM Ca^2+^ for 10 min then subjected to a 5 mM Ca^2+^ bolus (black bar). Fish used were WT, MCU OE, or MCU OE retinas preincubated in Ru360 (100 µM) and maintained throughout the experiment. *N* = 33 cells (seven fish) for WT, *n* = 31 cells (seven fish) for MCU OE, and *n* = 26 cells (three fish) for MCU OE + Ru360. Fish were between 3–5 months of age and slices were imaged every 2 s. The mean is reported and shaded region = standard error. **b** Decay constant of Ca^2+^ clearance for experiments shown in A. The mean is reported and bars = standard error. ***p < 0.001 using ANOVA followed by Tukey post hoc test. **c** Peak GCaMP3 fluorescence fold change from baseline for experiments shown in A. The median is reported and bars = interquartile range. WT: median = 2.14, Q1 = 1.87, and Q3 = 2.42. MCU OE: median = 1.68, Q1 = 1.46, and Q3 = 2.02. MCU OE + Ru360: median = 2.02, Q1 = 1.87, and Q3 = 2.80. **p < 0.01, ***p < 0.001 using Kruskal–Wallis followed by Dunn post hoc test. **d** The normalized ex vivo a-wave response isolated using DL-AP4 (40 µM) and CNQX (40 µM). Each retina response is normalized to *R*_max_, the maximum response at the brightest light intensity. Bright flash stimulus intensity is 800,457 photons µm^−2^ and 20 ms in duration. *N* = 19 retinas (11 fish) for WT siblings, *n* = 24 retinas (14 fish) for MCU OE. Fish were 7 months of age. The mean is reported and shaded region = standard error. **e** Time to half-maximum of the individual responses to a dim stimulus flash from data shown in D. Dim flash stimulus intensity is 2144 photons µm^−2^ and 5 ms in duration. The median is reported and bars = interquartile range. WT: median = 68.1 s, Q1 = 34.9, and Q3 = 132.4. MCU OE: median = 37.1 s, Q1 = 28.2, and Q3 = 63.0. *p < 0.05 using Mann–Whitney test

Cytosolic Ca^2+^ signals are critical for cone function, so we tested if MCU OE cones could clear cytosolic Ca^2+^ transients faster than in their WT counterparts. We preincubated retinal slices from *gnat2*:GCaMP3 fish in a 0 mM Ca^2+^ solution, then introduced a bolus of 5 mM CaCl_2_ and monitored clearance of cytosolic Ca^2+^ from the cell body (Fig. 6a). MCU OE cones clear Ca^2+^ from the cell body cytosol 2.3 ± 0.1 times faster than their WT siblings, as determined by the decay constant of a single exponential fit (Fig. 6b, Supplementary Fig. 6A). The peak fold change in cone cell body GCaMP3 fluorescence in response to the Ca^2+^ bolus is lower in MCU OE cones (Fig. 6c). To determine whether these changes were due to Ca^2+^ uptake via MCU, we incubated MCU OE retinal slices in the MCU inhibitor Ru360. Ru360 treatment partially but significantly restores the WT kinetics (Fig. 6a–c). The incompleteness of the effect may be attributed to other buffering mechanisms affected by MCU overexpression (such as the ER), insufficient permeability of Ru360 into cells, or an abundance of MICU1, which can block Ru360 binding to MCU [37].

Cytosolic Ca^2+^ in photoreceptor outer segments regulates the gain of phototransduction, light response recovery kinetics, and light adaptation [38–41]. Efficient clearance of Ca^2+^ from the outer segment in response to light is critical for rapid recovery of the photoresponse, so we asked whether overexpressing MCU in cones accelerates recovery prior to cone degeneration [4]. Using an ex vivo electroretinogram (ERG) technique to measure pharmacologically isolated cone photoreceptor responses, we found that the initial phase of photoresponse recovery following light flashes is accelerated by MCU overexpression (Fig. 6d). This is more apparent in the dim flash responses, which have a shorter time to half-maximum in MCU OE retinas (Fig. 6e). At the age tested (7 months) the maximal response amplitude (*R*_max_) is somewhat decreased in MCU OE retinas (Supplementary Fig. 6B). However, the dim flash responses normalized to *R*_max_ have larger amplitude in MCU cones compared with WT, suggesting an increased gain of phototransduction (Fig. 6d, Supplementary Fig. 6C). To evaluate this, we determined the amplification constant (*A*) using a model introduced by Lamb and Pugh [42]. We found a significant increase in *A* in MCU OE retinas, consistent with increased gain of phototransduction activation reactions (Supplementary Fig. 6D).

### Cones respond to MCU overexpression by decreasing MICU3 expression and selectively transporting abnormal mitochondria away from the ellipsoid

**Fig. 7:**
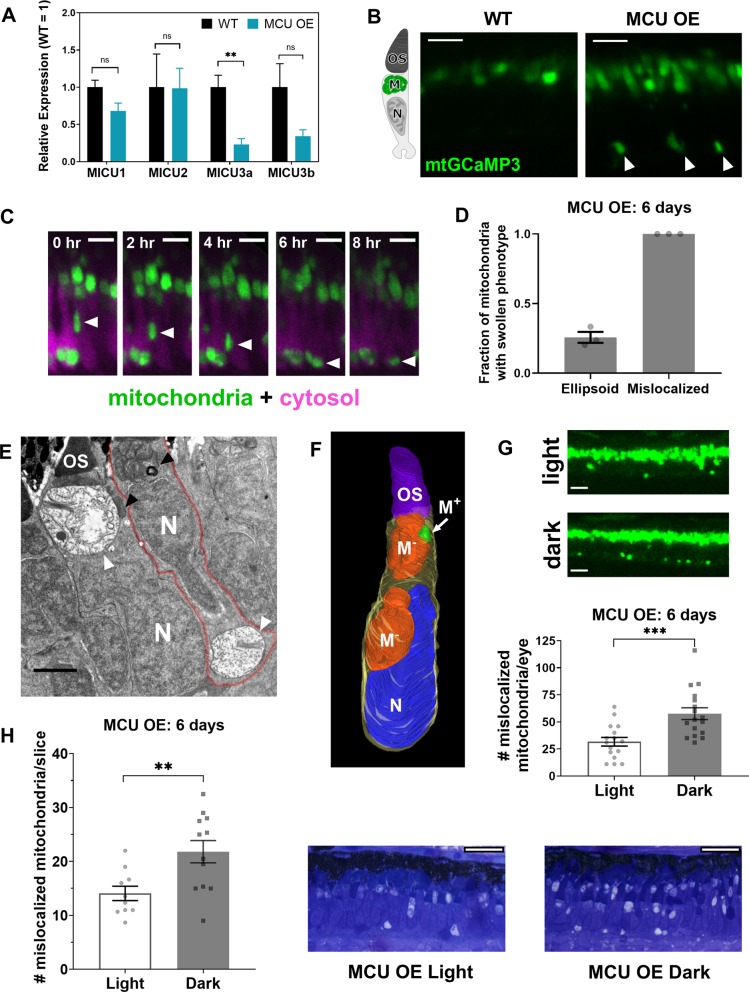
Cones respond to MCU overexpression by decreasing MICU3 transcript and selectively transporting abnormal mitochondria away from the ellipsoid. **a** qRT-PCR quantification of relative mRNA of MICU proteins (relative to reference gene *Ef1α*, see “Methods”) between WT and MCU OE retinas at 3 months of age (*n* = 3). The mean is reported and bars = standard error. **p < 0.01 and ns = not significant using Welch’s *t*-test. **b** Cone mitochondrial clusters in live larvae expressing *gnat2*:mito-GCaMP3. WT mitochondria were imaged with higher laser power to show localization. In MCU OE models, mitochondrial clusters were found near the synapse and nuclear layer (white arrows), which was not observed in WT siblings. Left: cone schematic with OS = outer segment, M = mitochondrial cluster, and N = nucleus. Scale bar = 5 µm. **c** 8 h timelapse of a migrating mitochondrial cluster (green, white arrow) in live MCU OE *gnat2*:mito-GCaMP3 larvae. Cone cell bodies express cytosolic RFP (magenta). Scale bar = 5 µm. **d** Quantification of cone mitochondria in WT sibling and MCU OE fish from EM images of whole zebrafish larval eyes (single slice at optic nerve) at 6 days of age. Fraction of swollen mitochondria was determined by counting swollen mitochondria relative to total cone mitochondria either in the ellipsoid or mislocalized. *n* = 3 larvae for both WT and MCU OE fish. The mean is reported and bars = standard error. **e** EM image from MCU OE larvae at 14 days of age. A single cone photoreceptor can contain both healthy mitochondria (black arrows) in the ellipsoid region and swollen mitochondria (white arrows) near the synapse. Cone cell membrane outlined in red overlay to aid visualization. Scale bar = 2 µm. OS = outer segment, N = nucleus. **f** 3D reconstruction of an MCU OE cone from a 6-day-old fish using serial block-face EM (synapse not shown). Electron-lucent mitochondria (M^−^) displace the nucleus (N) of the cone to move toward the synapse region. OS = outer segment. M^−^ = electron-lucent mitochondria. M^+^ = healthy mitochondria. N = nucleus. Outline (yellow) = cell body. **g** Quantification of mislocalized mitochondria using the mito-GCaMP3 probe in 6-day-old MCU OE fish under a normal light cycle or subjected to constant darkness at 5 days of age. Quantification was performed across a whole eye at a total depth of 50 µm. Scale bar = 10 µm. ***p < 0.001 using Welch’s *t*-test (*n* = 16 fish each). **h** Quantification of mislocalized mitochondria in Richardsons’-stained sections of 6-day-old fish under a normal light cycle or subjected to constant darkness at 5 days of age. Quantification was performed in 2–3 slices per eye at/near the optic nerve and the average # of mitochondria for each eye is reported. Scale bar = 25 µm. **p < 0.01 using Welch’s *t*-test (*n* = 10 eyes in light, 12 eyes in dark)

We assessed what adaptations may enable long-term survival of MCU OE cones. We analyzed retinal transcripts of MICU1, MICU2, MICU3a, and MICU3b in 3-month-old retinas and found that MICU3a, reported to be a neuronal-specific enhancer of Ca^2+^ uptake via MCU [18], was significantly lower in MCU OE retinas (Fig. 7a). This would presumably limit Ca^2+^ influx into mitochondria and thereby contribute to cone survival. However, this cannot compensate completely for the MCU overexpression phenotype since we observe swollen mitochondria and increased basal mitochondrial Ca^2+^ at this age and beyond (Figs. 3c, 4e, Supplementary Fig. 4B).

Cones also respond to MCU overexpression by altering their mitochondrial distribution. Mitochondria normally are confined solely to the ellipsoid region in zebrafish cones, between the nucleus and the outer segment [43]. However, large mitochondrial clusters are present outside of this region in MCU OE retinas (Fig. 7b). We recorded time lapses of live zebrafish larvae expressing *gnat2*:mito-GCaMP3 and *gnat2*:MCU-T2A-RFP that revealed a slow, directed movement of mitochondrial clusters away from the ellipsoid region toward the synapse (Fig. 7c).

All mitochondria observed outside of the ellipsoid region of MCU OE cones have a swollen phenotype by electron microscopy (Fig. 7d). Some MCU cones with swollen, mislocalized mitochondria contain mostly normal mitochondria within the ellipsoid (Fig. 7e). The 3D reconstructions of MCU OE mitochondria from electron micrographs suggest that movement of abnormal mitochondria away from the ellipsoid region is active, as moving mitochondria deform the nucleus on their way toward the synapse (Fig. 7f, Supplemental Video 1).

These observations led us to hypothesize that cones can selectively export damaged mitochondria from the ellipsoid and that Ca^2+^ may stimulate this. If this were true, subjecting MCU OE cones to higher intracellular Ca^2+^ would increase Ca^2+^-associated mitochondrial stress and mitochondrial movement. Intracellular Ca^2+^ in cones is constitutively elevated in darkness, so we compared MCU OE fish on a normal light cycle with those incubated in complete darkness the day prior to imaging. Dark preincubation increases the number of mislocalized mitochondria in *gnat2*:mito-GCaMP3 fish (Fig. 7g). This is not attributable to changes in probe fluorescence, which was unchanged under imaging conditions (Dark = 103 ± 5% of Light, *p* = 0.653 using Welch’s *t*-test, *n* = 16 fish). Quantification of mislocalized mitochondria in Richardson’s stained sections confirms that darkness exposure increases mitochondrial mislocalization (Fig. 7h).

## Discussion

**Fig. 8:**
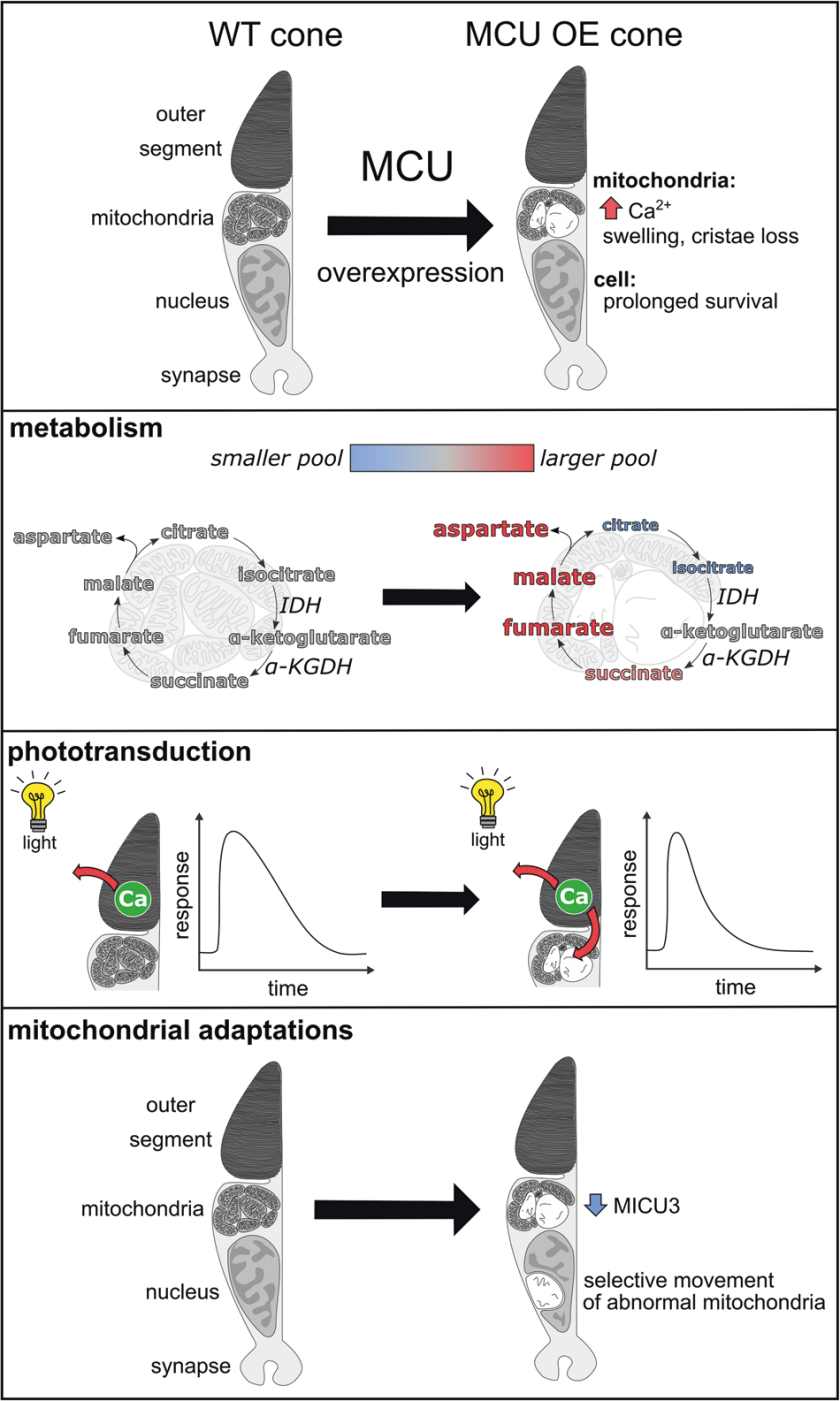
Summary of phenotypes in the MCU OE cone model. Cones, which normally have low MCU expression, experience an increase in mitochondrial matrix Ca^2+^ and mitochondrial swelling when MCU is overexpressed. Cones survive this stress for many months. Mitochondrial metabolite pools are altered consistent with enhanced activity of IDH and α-KGDH. Recovery of the photoreceptor to light stimuli, dependent on Ca^2+^ clearance from the outer segment, is faster when MCU is overexpressed. Cones respond to chronic elevated mitochondrial Ca^2+^ by decreasing transcription of the MCU enhancer MICU3 and selectively moving abnormal mitochondria away from the ellipsoid region of the cell

The key findings of our study are (1) MCU is expressed at very low levels in cone photoreceptors, (2) metabolic and physiologic functions of cones are influenced by enhanced mitochondrial Ca^2+^ influx, and (3) cones can adapt to elevated mitochondrial Ca^2+^ and survive this stress for many months. These findings are summarized in Fig. 8.

Our findings challenge the idea that mitochondrial Ca^2+^ overload is a primary driver of photoreceptor degeneration. MCU in cones is normally maintained at low levels, yet cones can robustly survive MCU expressed beyond physiological levels that causes a fourfold increase in basal matrix [Ca^2+^] and mitochondrial disruption. Their prolonged survival suggests mitochondrial Ca^2+^ overload may not be the cause of rapid photoreceptor degeneration linked to mutations that increase cytosolic [Ca^2+^]. This is consistent with the finding that cellular Ca^2+^ buffering is not overloaded in zebrafish pde6c mutant cones or rd1 mutant mouse rods [44]. Our findings appear to differ from an ex vivo study showing mitochondria-mediated cell death in rods caused by elevated external Ca^2+^ [6]. A possible explanation for these different findings is that our study monitors cell survival within a living organism, where the stress is chronic and normal adaptive mechanisms are intact.

### Cone adaptations to mitochondrial Ca^2+^ stress

Determining how cones survive chronic mitochondrial Ca^2+^ stress and mitochondrial disruption is key to understanding cone homeostasis and long-term viability. We found that cones respond to elevated mitochondrial Ca^2+^ by decreasing expression of the Ca^2+^ uptake enhancer MICU3 and selectively transporting abnormal mitochondria away from the ellipsoid. Mitochondrial movement away from the ellipsoid could protect cones from mitochondrial Ca^2+^ overload. The ellipsoid is adjacent to the outer segment, which reaches high, sustained levels of Ca^2+^ in darkness. Trafficking of damaged mitochondria may be an important adaptation for long-term survival of normal cones; cones in aging humans contain swollen mitochondria similar to MCU OE mitochondria, and retinal mitolysosomes are highly concentrated in the photoreceptor nuclear layer, away from the ellipsoid [45, 46]. We hypothesize that selective mitochondrial movement can occur in normal cones, but is dramatically enhanced by the widespread mitochondrial damage upon MCU overexpression.

Both cytosolic and mitochondrial matrix Ca^2+^ are associated with reduced mitochondrial movement in neurons [47–50]. Mitochondrial damage is also associated with cessation of movement [51–53]. However, in MCU OE cones damaged mitochondria are selectively trafficked, and higher intracellular Ca^2+^ in darkness enhances this movement. It is possible that acute mitochondrial stress responses differ from chronic stress responses, which have recently been reported to trigger release of the anchoring protein SNPH to induce transport of stressed mitochondria [54]. These disparate observations highlight the need for further investigation of the role of mitochondrial Ca^2+^ content and subsequent stress in mitochondrial movement in neurons.

### Mitochondria can alter phototransduction kinetics

Mitochondria can act as a barrier between the Ca^2+^ pools in the outer segment and the rest of the cell [9]. Their proximity to the outer segment raises the possibility that they influence Ca^2+^ in the outer segment, where Ca^2+^ clearance is essential to promote photoresponse recovery and adaptation. Clearance of outer segment Ca^2+^ is thought to be accomplished primarily by plasma membrane Na^+^/Ca^2+^, K^+^ exchangers, but cones lacking these can respond to light, light adapt, and degenerate rather slowly, suggesting that there is an additional pathway that clears Ca^2+^ from cone outer segments [55, 56]. Survival of MCU OE cones allowed us to investigate the capacity for mitochondria to influence the photoresponse, and we report that faster clearance of cytosolic Ca^2+^ is accompanied by accelerated photoresponse recovery. This indicates that mitochondrial Ca^2+^ uptake in photoreceptors can contribute to Ca^2+^ clearance from the outer segment and modulate the kinetics of the photoreceptor response to light. Mitochondria may contribute to normal photorecovery, as delayed rod photorecovery is observed in human patients with malfunctioning mitochondria [57].

### MCU overexpression enhances IDH and α-KGDH activity

The perturbations to mitochondrial structure and localization in MCU OE cones could cause Ca^2+^-independent changes to mitochondrial metabolism. However, TCA cycle metabolites are not globally decreased and MCU OE retinas have unaltered glycolytic activity, suggesting they are not compensating for decreased mitochondrial metabolic function. Instead of disrupting mitochondrial metabolism, MCU overexpression specifically alters the steady-state levels of TCA cycle metabolites, consistent with enhanced activities of the Ca^2+^-sensitive enzymes IDH and α-KGDH.

The highest levels of intracellular free Ca^2+^ in photoreceptors occur in darkness, when energy demand and O_2_ consumption are greatest. Ca^2+^ could play an important role in stimulating increased TCA cycle activity in photoreceptors in darkness [58, 59]. Rod photoreceptors in mouse retinas accumulate higher levels of TCA cycle metabolites downstream of α-KGDH in darkness than in light [60]. Here, we report a similar accumulation of downstream metabolites in MCU OE cones. We also note that enhanced IDH and α-KGDH activities deplete upstream pools of citrate and isocitrate. MCU OE retinas do not increase citrate production in response to a decrease in the steady-state level of citrate, suggesting that TCA cycle activity in cones is limited either by pyruvate entry into mitochondria or by acetyl-CoA production.

An increase in the P-PDH/total PDH ratio is a common metabolic phenotype in MCU KO tissues [61]. However, we did not find evidence for Ca^2+^ stimulation of PDH phosphatase in cones. Since the P-PDH/total PDH ratio also does not decrease when MCU is overexpressed in muscle cells, it is possible that changes in mitochondrial bioenergetics resulting from increased mitochondrial Ca^2+^ feed into the complex regulation of PDH [27]. For example, stimulation of α-KGDH and IDH activity may result in higher NADH levels in MCU OE cones, which in turn stimulates PDH kinase to balance increased PDP1c activity.

### Conclusions and future directions

Photoreceptors must maintain viability throughout a lifetime despite chronic stress associated with light damage, ATP demand, O_2_ exposure, and Ca^2+^ fluctuations. Our findings show that cone photoreceptors are remarkably tolerant of Ca^2+^-associated mitochondrial stress, an adaptation that likely promotes their long-term survival. Cones can survive high levels of MCU expression, and while excess protein could potentially cause other secondary effects, we observe specific changes to metabolism and phototransduction kinetics consistent with enhanced mitochondrial Ca^2+^ uptake. Cones respond to elevated mitochondrial Ca^2+^ by selectively trafficking abnormal mitochondria away from their normal position in the ellipsoid and decreasing transcription of the Ca^2+^ uptake enhancer MICU3. The cone MCU OE model can be used in future studies as a tool to examine mitochondrial movement and trafficking in cones, and dissect in more detail the adaptive mechanisms that promote survival in response to chronic mitochondrial stress. Furthermore, the distinct localization of mitochondria in zebrafish cones makes the MCU OE model an attractive system to study mechanisms underlying mitochondrial sorting and movement.

## Materials and methods

### Zebrafish maintenance

Experiments with zebrafish were authorized by the University of Washington and University of Utah Institutional Animal Care and Use Committees. All fish used in this analysis were maintained in the University of Washington South Lake Union aquatics facility or the Centralized Zebrafish Animal Resource (CZAR) at the University of Utah at 27.5 °C on a 14/10 h light/dark cycle, and were maintained in the Roy^−/−^ genetic background. All wild-type fish (WT) used in analysis were age-matched siblings to Tg(gnat2:MCU-T2A-RFP) fish (MCU OE) or age-matched siblings to pde6c^w59^ (pde6c^−/−^) [19]. Fish used for slice preparation, protein quantification, and metabolomics analysis were male and female siblings between 3 and 6 months of age. Fish used in ERG analysis were male and female siblings collected at 7 months of age. For histological analysis, ages of sibling fish are included in the figure and legend. Formal methods of randomization were not used.

### Zebrafish MCU antibody

The cDNA encoding amino acids 21–202 of *Danio rerio* MCU (NM_001077325) was cloned downstream of GST using the pGEX-2T (GE) expression vector. Overexpression was induced in *E.coli* (BL21) by addition of 1 mM IPTG at 0.2 OD followed by incubation with vigorous shaking for 5 h at 37 °C. The tiny fraction of soluble fusion was purified using glutathione sepharose following the manufacturer’s instructions (GE Healthcare). Polyclonal antibodies were generated using injections of 0.5–1 mg protein (R and R Research Co.). Two columns were used to clean the serum. One column contained total *E.coli* proteins covalently coupled to cyanogen bromide beads and the second column contained purified GST protein coupled to cyanogen bromide beads (GE Healthcare). Serum was cleaned by sequential incubations of 3–5 h at room temperature with each column after which it was analyzed on an SDS page gel for lack of cross reactivity with GST and *E.coli* proteins from a total cell extract. Identification of MCU was validated by the absence of a protein of the correct molecular weight in extracts obtained from a CRISPR generated KO strain. The zebrafish MCU antibody was used at a dilution of 1:750 for western blotting and 1:50 for immunohistochemistry (IHC).

### Zebrafish models

The transgenic zebrafish lines Tg(gnat2:GCaMP3), Tg(gnat2:EGFP), and Tg(mito-GCaMP3) have been described previously [9, 44, 62]. Generation of the global MCU KO line was performed using gRNA with the following sequence 5′-CCTCATACCTGGTGCAGCCCCCC-3′ using methods as previously described [63]. For generation of the Tg(*gnat2*:MCU-T2A-RFP) line, zebrafish MCU cDNA was isolated from WT zebrafish larvae (5 dpf) using the forward primer 5′-AGAGATGGCTGCGAAAAGTGT-3′ and reverse primer 5′-TTCTCATCAGTCCTTGCTGGT-3′. Overhang qPCR methods in conjunction with Fast Cloning were used to add the T2A ribosomal stalling sequence and the RFP protein coding sequence; this was cloned into a pCR8/GW vector (Invitrogen). Plasmids were assembled using the Gateway-Tol2 system [64]. Expression of MCU–T2A–RFP was driven by the cone transducin alpha promoter (TαCP, gnat2), and the RFP coding sequence was flanked by a polyA tail sequence to increase transcript stability [62]. A destination vector with a sBFP2 heart marker for aid in transgenic identification was obtained from Cecilia Moens [65]. The fully assembled construct was injected into embryos at the 1-cell stage with Tol2 transposase mRNA. Larvae mosaic for the transgene were raised to adulthood to identify founder carriers. A single *F*_0_ founder was used to generate *F*_1_ fish that were screened for a single insertion of the transgene; *F*_2_ fish from two *F*_1_ substrains with a single insertion were used for analysis in this study.

### Primers for qRT-PCR

All designed primers were empirically tested to confirm primer efficiency was between 90 and 110%. Only primers passing this benchmark were used for analysis. Primer sequences for the reference genes *EF1a*, *b2m*, *Rpl13a*, and *TBF* were identical to previous reports testing zebrafish reference gene stability (*EF1a*, *Rpl13a*:ref. 66, *b2m*, *TBP*:ref. 67).

MICU1:

Forward: 5′-ACGTTAAAGCAGAATCGTAGAGG-3′

Reverse: 5′-CGCAAGCGGTACATATCAGAC-3′

MICU2:

Forward: 5′-ACTGAGTACCTGTTTCTCCTCAC-3′

Reverse: 5′-GGTCCATTTACTTTCTTCAGCTTCT-3′

MICU3a:

Forward: 5′-CGTCCCATGAGCATCGTTTC-3′

Reverse: 5′-TCCAACTCCTGTTTGGTGAGG-3′

MICU3b:

Forward: 5′-GCTTGGTGCAAGAATAGTTCTCTTT-3′

Reverse: 5′-TGCAGGTTGTCCATGAATCTGT-3′

### qRT-PCR

An Applied Biosystems 7500 Fast Real-Time PCR System in conjunction with iTaq™ Universal SYBR^®^ Green Supermix (Bio-Rad, 1725120) was used for qPCR measurements according to the manufacturer’s instructions. The reference genes *EF1a*, *b2m*, *TBF*, and *rpl13a* were screened across the tissue panel using NormFinder to identify reference genes with the highest stability [68]. NormFinder identified the combination of *EF1a* and *b2m* as most stable for retina–brain comparisons and *EF1a* as most stable for retina–heart comparisons. *EF1a* was identified as the most stable for WT vs MCU OE retina comparisons. Quantification of relative mRNA quantity used three biological replicates of each tissue, each performed in technical triplicate. From each technical triplicate, the average *C*_t_ value for the gene of interest and reference gene(s) were used to generate a Δ*C*_t_ value for each biological replicate. Comparing each tissue of interest with the retina generated a ΔΔ*C*_t_ value; these were converted to a normalized expression level using the ${2}^{{-\Delta\Delta}{C}_{\rm{t}}}$ method (Livak assumptions). Standard error of the Δ*C*_t_ value for each tissue was propagated to the final comparison using standard error propagation rules. Calculations were based off the geNorm method of qPCR normalization [69].

### Commercial antibodies and stains

MTCO1 (Abcam, ab14705, RRID:AB_2084810); used in IHC and immunoblotting at 1:1000 dilution. SDHB (Abcam, ab14714, RRID:AB_301432); used in IHC and immunoblotting at 1:1000 dilution. PDH E1 subunit, PDH (Abcam, ab110334, RRID:AB_10866116)**; u**sed in immunoblotting at 1:1000 dilution. Phosphorylated PDH E1 subunit Ser293, P-PDH (EMD Millipore, ABS204, RRID:AB_11205754); used in immunoblotting at 1:2000 dilution. Pyruvate Kinase, PK (Abcam, ab137791); used in immunoblotting at 1:1000 dilution. Hoechst 33,342, Trihydrochloride, Trihydrate stain (ThermoFischer, H3570); used in IHC at 5 µM concentration. Lectin PNA Alexa Fluor 647 conjugate (ThermoFischer, L32460); used in IHC at 1:200 dilution after suspending at a concentration of 1 mg/mL in H2O. Goat Anti-Mouse IgG H&L, Alexa Fluor 488 (Abcam, ab150113, RRID:AB_2576208); used in IHC at 1:1000 dilution. Goat Anti-Rabbit IgG H&L, Alexa Fluor 647 (Abcam, ab150083, RRID:AB_2714032); used in IHC at 1:1000 dilution. IRDye 800CW donkey anti-rabbit IgG (H + L) (LI-COR Biosciences, 925-32213, RRID: AB_2715510); used at 1:5000 dilution for immunoblotting. IRDye 680RD donkey anti-mouse IgG (H + L) (LI-COR Biosciences, 925-32212, RRID: AB_2716622); used at 1:5000 dilution for immunoblotting. IRDye 680RD donkey anti-rabbit IgG (H + L) (LI-COR Biosciences, 925-68073, RRID: AB_2716687); used at 1:5000 dilution for immunoblotting. IRDye 800CW goat anti-mouse IgG (H + L) (LI-COR Biosciences, 925-32210, RRID: AB_2687825); used at 1:5000 dilution for immunoblotting.

### Mitochondrial enrichment and sample preparation for immunoblotting

Freshly dissected organs were homogenized with a dounce homogenizer in 50 mM Tris buffer containing sucrose (200 mM), NaCl (150 mM), and EGTA (1 mM) with a protease inhibitor mini tablet (ThermoFischer, 88666). Homogenized samples were centrifuged at a low speed of 1000 × *g* for 10 min at 4 °C, then the supernatant (containing mitochondria) was collected and centrifuged at a high speed of 17,000 × *g* for 45 min. The supernatant was discarded and the pellet (containing mitochondria) was homogenized for 1 min in RIPA buffer. Homogenized mitochondria were sonicated on ice for three 5 s pulses. A standard BCA assay using Pierce™ BCA Protein Assay Kit (ThermoFischer, 23225) was performed according to the manufacturer’s instructions for protein concentration determination. Samples were diluted with RIPA buffer to ensure an equal volume and equal protein concentration of each sample could be loaded into wells for immunoblotting.

### Immunoblotting

Samples were loaded into wells on 12–14% acrylamide gels made in house. Each sample contained 20% 5× SDS buffer containing β-mercaptoethanol. After running the gel at 150 V for 1 h, gels were transferred onto PVDF membranes (Millipore, IPFL00010) and blocked for 1 h at room temperature in LI-COR Odyssey Blocking Buffer (LI-COR, 927–40,000). Primary antibodies were diluted in blocking buffer at specified concentrations and incubated overnight at 4 °C. Membranes were washed with PBST and PBS, then incubated with secondary antibody for 1 h at 25 °C and washed again before imaging. Membranes were imaged and bands were quantified using the LI-COR Odyssey CL× Imaging System (RRID:SCR_014579). Gels were repeated a minimum of twice, with images from one representative experiment.

### Immunohistochemistry (IHC) and degeneration quantification

All adult eyes were isolated from light-adapted zebrafish, and a small incision in the cornea was made to allow 4% paraformaldehyde fixative to enter the eye. Whole larvae were euthanized then incubated in 4% paraformaldehyde. After fixation overnight at 4 °C, eyes were rinsed in PBS then subject to a sucrose gradient (20 and 30%), embedding in OCT, and cryosectioned at 12 µm. For sections stained with MCU antibody, antigen retrieval was performed by steaming sections in 10 mM sodium citrate (0.05% Tween-20, pH 6.0). Sections were washed in PBS, then blocked in PBS containing 5% donkey serum, 2 mg/mL bovine serum albumin, and 0.3% Triton X-100 for 1 h. Primary antibodies were diluted in this buffer as specified, then applied to cryosection overnight at 4 °C. Secondary antibodies were diluted as specified and applied to section for 1 h in darkness at 25 °C. For PNA-labelled samples, sections were incubated in diluted PNA-647 for 30 min at 25 °C. Tissues were washed, incubated in Hoechst stain for 10 min, and then mounted in Fluoromount-G^®^ (SouthernBiotech, 0100-01) under glass coverslips. Slides were imaged using a Leica LSP8 confocal microscope with a 63X oil objective. Leica LAS-X software (RRID:SCR_013673) was used to acquire images.

For quantification of cone nuclei in *gnat2*:GFP fish, high-resolution images of whole zebrafish retina slices were stitched together using ImageJ Grid/Collection stitching [70]. Both the dorsal and ventral regions of the retina were straightened along the cone nuclei axis using ImageJ from the optic nerve to the cilliary margin. This axis was divided into five equal parts, then double-cone nuclei were counted in each region, normalizing to the length in micrometre (height of the region was equal across samples, double cone nuclei are along a single axis). Double-cone nuclei were used for quantification as they are most easily distinguished from rod nuclei. GFP expression was used to confirm that the double-cone nuclei counted were indeed cone nuclei. All counting was performed blinded (masked) to sample identity.

### Live larval imaging of mito-GCaMP3

Larvae used for imaging were maintained in embryo media containing 0.003% 1-phenyl 2-thiourea (PTU, Sigma-Aldrich P7629) starting at 20 h postfertilization. Live zebrafish larvae were analyzed at 6 days postfertilization (dpf) by transferring to 0.5% low melting point agarose containing embryo media with 0.003% PTU and 0.02% (w/v) Tricaine (Sigma-Aldrich, E10521). Larvae were positioned in agarose in a petri dish containing embryo media and 0.02% (w/v) tricaine to prevent drying out. Imaging of slices was performed using an Olympus FV1000 in conjunction with Olympus FluoView FV10-ASW software (RRID:SCR_014215). A 40× water objective was used for imaging. The excitation/emission wavelengths used for mito-GCaMP3 were 488/510 nm. Timelapse images of live larvae were collected with a z-depth of 2 µm and were collected every 20 min. Images of total eye mitochondrial clusters were also collected at a z-depth of 2 µm. For quantification of total mito-GCaMP3 fluorescence, images of whole larval eyes were collected, and a fixed ROI centered on the nasal region of the retina was used for quantification. This region near the ventronasal patch is comprised of the most mature cone photoreceptors [71]. For quantification of mislocalized mitochondria, laser power was increased to saturate signals and allow visualization of all mitochondria. Stacks of 50 µm in depth were collected across the entire eye for quantification and mitochondrial clusters were counted blinded (masked) to sample identity. Larval experiments were repeated across at least two cohorts of siblings, with images and quantification from a representative experiment.

### Retinal slice imaging of GCaMP3 and mito-GCaMP3

Slices were prepared as described previously [9, 72]. For GCaMP3 cytosolic clearance experiments, slides were preincubated in KRB containing 0 mM Ca^2+^ and 0.4 mM EGTA for 10 min. Images of single optical slices were collected every 2 s. A 5 mM CaCl_2_ bolus (accounting for EGTA) was injected into the slice imaging chamber 30 s after the initial timelapse collection to establish baseline fluorescence. Injection volume was 1 mL into the 4 mL imaging chamber, mixed thoroughly. These experiments in the MCU-overexpressing fish were additionally performed in the presence of Ru360 (Millipore, 557440) at 100 µM, in which slices were incubated for 1 h prior to incubation in 0 mM Ca^2+^ media. Retinas treated with Ru360 were maintained in Ru360 throughout timelapse experiments. Dying cells near the cut edge that were constitutively loaded with Ca^2+^ and cells that did not respond to Ca^2+^ were not included in analysis.

For mito-GCaMP3 timelapse experiments, z-stacks of 15, 2 µm slices were collected every 30 s. Retinal slices in modified KRB containing 2 mM CaCl_2_ were first imaged for 5 min to establish baseline mito-GCaMP3 fluorescence. Next, the chamber was injected with ionomycin (Sigma, 407950) to a final concentration of 5 µM (prepared in DMSO, at a final concentration of 0.1%) for another 5 min of image collection. Finally, an excess of EGTA (5 mM) was injected to chelate the 2 mM Ca^2+^ present in solution and images were collected for another 5 min. Injection volume was 1 mL into the 4 mL imaging chamber, mixed thoroughly. Dying cells containing fragmented mitochondrial clusters constitutively loaded with Ca^2+^ and clusters that did not respond to ionomycin were not included in analysis. In addition, any clusters where the maximum fluorescence signal in the presence of ionomycin was completely saturated were excluded from analysis. Analysis was conducted blinded (masked) to sample identity.

The excitation/emission wavelengths used for both GCaMP3 and mito-GCaMP3 were 488/510 nm. Timelapses were analyzed using ImageJ + Fiji software (SCR_002285). Images were corrected for X-Y drift using the MultiStackReg plugin of ImageJ. For both cell body GCaMP3 and mito-GCaMP3 fluorescence ex vivo timelapses, fixed ROIs were used to quantify average fluorescence signal across the cluster/cell at every time point. Fluorescence of cytosolic GCaMP3 for timelapse analysis are reported as *F*/*F*_0_, where *F*_0_ is the baseline fluorescence. For mito-GCaMP3 fluorescence, the relative fluorescence at maximum was set to 100% for normalization. We used the equation $\left[ {Ca^{2 + }} \right] = K_D \times \frac{\theta }{{1 - \theta }}$, where $\theta = \frac{{F_0 - F_{min}}}{{F_{max} - F_{min}}}$ to approximate [Ca^2+^]_mito_, where *F*_0_ is the average “baseline” fluorescence, *F*_max_ is maximum fluorescence upon ionomycin addition, and *F*_min_ is the baseline fluorescence upon EGTA addition. We approximated the *K*_D_ of GCaMP3 at 345 nM for the calculation [23].

### Electroretinograms (ERG)

Zebrafish were briefly dark adapted (~30 min), before euthanasia by ice water immersion. Eyes were enucleated into Modified Salamander Ringer’s solution(mM): NaCl 110, KCl 2.5, CaCl_2_ 1.0, MgCl_2_ 1.6, HEPES 10.0, and Glucose 10.0, with pH adjusted to 7.8 with NaOH. The eyes were hemisected and retinas isolated from the eyecup. All procedures after the dark adaptation were performed under dim red light. To ensure ex vivo ERG signal was predominantly cone responses, dark adaption was limited to ~30 min to allow cone photopigment regeneration but not provide enough time for full rod photopigment regeneration. Furthermore, experiments were carried out during the day (between 11a.m. and 4p.m.), when rod contributions to retinal responses are at their lowest due to the circadian regulation of photoreceptor biology in the zebrafish retina. Ex vivo ERG recordings were performed as described previously [73]. Isolated retinas were mounted photoreceptor side up onto the specimen holder [74], and perfused with Modified Salamander Ringer’s solution, supplemented with 40 µM DL-AP4 (Tocris Bioscience) and 40 µM CNQX (Tocris Bioscience) to isolate the photoreceptor component of the ERG signal (a-wave). The rate of perfusion was ~5 mL/min and the experiments were conducted at room temperature (~23 °C).

ERG signal was first amplified (100×) and low-pass filtered at 300 Hz by a differential amplifier (DP-311, Warner Instruments), and data was further amplified (10×) and acquired at 10KHz using an integrated amplifier/digitizer (IPA, Sutter Instrument, CA). A High Power LED light source (Solis-3C, Thorlabs, Newton, NJ), with filter for red light (630 nm, FWHM bandwidth 69 nm, FF01-630/69-25, Semrock, Rochester, NY) and LED driver (DC2200, Thorlabs) were used to provide the flashes of light stimuli, durations ranged from 5 to 100 ms. The SutterPatch software (SutterPatch v1.1.2, Sutter Instrument, CA) drove both stimulus generation and data acquisition via the IPA amplifier’s analogue output and input, respectively. Light stimuli were calibrated before experiments using a calibrated photodiode (FDS100-CAL, Thorlabs, Newton, NJ) and flash intensities converted to photons/µm^2^.

Data analysis, including statistical analysis and figure preparation, was performed with GraphPad v 8.00 (for Windows, GraphPad Software, CA, USA). Normalized responses were calculated for each retina by dividing the response amplitude data by the maximal amplitude measured at the peak/plateau of the response to the brightest flash. To quantify the gain of phototransduction activation, we fitted the Lamb–Pugh model to the initial leading edge of the dim flash response for each retina, and compared the average amplification constant (*A*) between WT and OE siblings [42].

### Isotopic labeling and mass spectrometry

Krebs-Ringer bicarbonate (KRB) buffer optimized for metabolic analysis was used in these experiments [75]. Zebrafish retinas were first dissected in KRB buffer containing U-^12^C-glucose or U-^12^C-glutamine at the same concentration they would be incubated in. After dissection, retinas were placed in dishes of prewarmed KRB containing either U-^13^C glucose (5 mM, Cambridge Isotopes, CLM-1396) or U-^13^C glutamine (0.1–2 mM, Cambridge Isotopes, CLM-1822). Retinas were incubated in this solution for the specified time points at 28 °C in a NAPCO Series 8000 WT CO_2_ incubator (5% CO_2_), then washed in ice-cold PBS and flash frozen in liquid nitrogen. Metabolites were extracted using ice-cold 80% MeOH and lyophilized. Two-step derivatization was performed by the addition of 20 mg/mL Methoxyamine HCl dissolved in pyridine, followed by *tert*-butyldimethylsilyl. Metabolites were analyzed on an Agilent 7890/5975C GC–MS as described extensively in previous work [60, 75–77]. Metabolic flux experiments were repeated a minimum of twice, using three retinas from three different zebrafish for each condition in each experiment. Data shown are results from one representative experiment.

### Electron microscopy and Richardson’s staining

Adult zebrafish eyes were enucleated and a small incision was made in the cornea to allow fixative (4% glutaraldehyde in 0.1 M sodium cacodylate buffer, pH 7.2) to enter the eye. Tissues were stored at 4 °C before postfixation in osmium ferrocyanide (2% osmium tetroxide/3% potassium ferrocyanide in buffer) for 1 h, followed by incubation in 1% thiocarbohydrazide for 20 min. Samples were then incubated in 2% osmium tetroxide for 30 min at RT, and stained with 1% aqueous uranyl acetate overnight at 4 °C. Samples were next stained en bloc with Walton’s lead aspartate for 30 min at 60 °C, dehydrated in a graded ethanol series, and embedded in Durcupan resin. Sections of tissue were cut at 60 nm thickness and imaged using a JEOL JEM-1230 transmission electron microscope or Zeiss Sigma VP scanning electron microscope. Samples of larval zebrafish eyes were imaged in conjunction with a Gatan 3View2XP ultamicrotome apparatus to generate stacks of EM images, which were aligned using TrakEM2 software (RRID:SCR_008954). Position in the eye for EM imaging was confirmed by cutting slices of tissue and staining with Richardson’s stain [78]. These slices were imaged for histological analysis using a Nikon Eclipse E1000 with a Nikon Plan Apo 100×/1.40 DIC lens. Nikon ACT-1 software was used for image capture. For quantification of mislocalized mitochondria, multiple sections were cut and imaged for each eye and the average number of mitochondria outside of the ellipsoid is reported for each eye. All counting was performed blinded (masked) to sample identity.

### Statistics

Numerical results in text are reported as mean ± standard error of the mean unless otherwise stated. Statistical tests were performed using Graphpad Prism v 8.00 software. For statistical analysis, replicates (*n*) were always defined as biological replicates. Information on what constitutes *n* (e.g., larvae, retinas, and cells) is listed in the figure legend of each experiment. Samples sizes were estimated based on previous experiments [9, 41, 60]. For data sets with sufficient *n* to analyze population distribution, tests for normality were administered (Anderson–Darling, D’Agostino & Pearson, Shapiro–Wilk, Kolmogorov–Smirnov). For data sets that did not pass a majority of normality tests, the median is instead reported along with the interquartile range (Q1 and Q3).

## Supplementary information

Supplemental Figure 4: Further characterization of retinal health throughout development in both WT and MCU OE models

Supplemental Figure 5: Further metabolic characterization of MCU OE retinas

Supplemental Figure 6: Fitting of Ca2+ clearance data and other ERG parameters

Supplemental Video 1

Supplementary figure legends
