# Supplementary figures and images for: Increasing Ca2+ in photoreceptor mitochondria alters metabolites, accelerates photoresponse recovery, and reveals adaptations to mitochondrial stress
Source: Cell Death Differ. 2019 Aug 2;27(3):1067–85. doi: 10.1038/s41418-019-0398-2 (PMC7206026; doi:10.1038/s41418-019-0398-2)

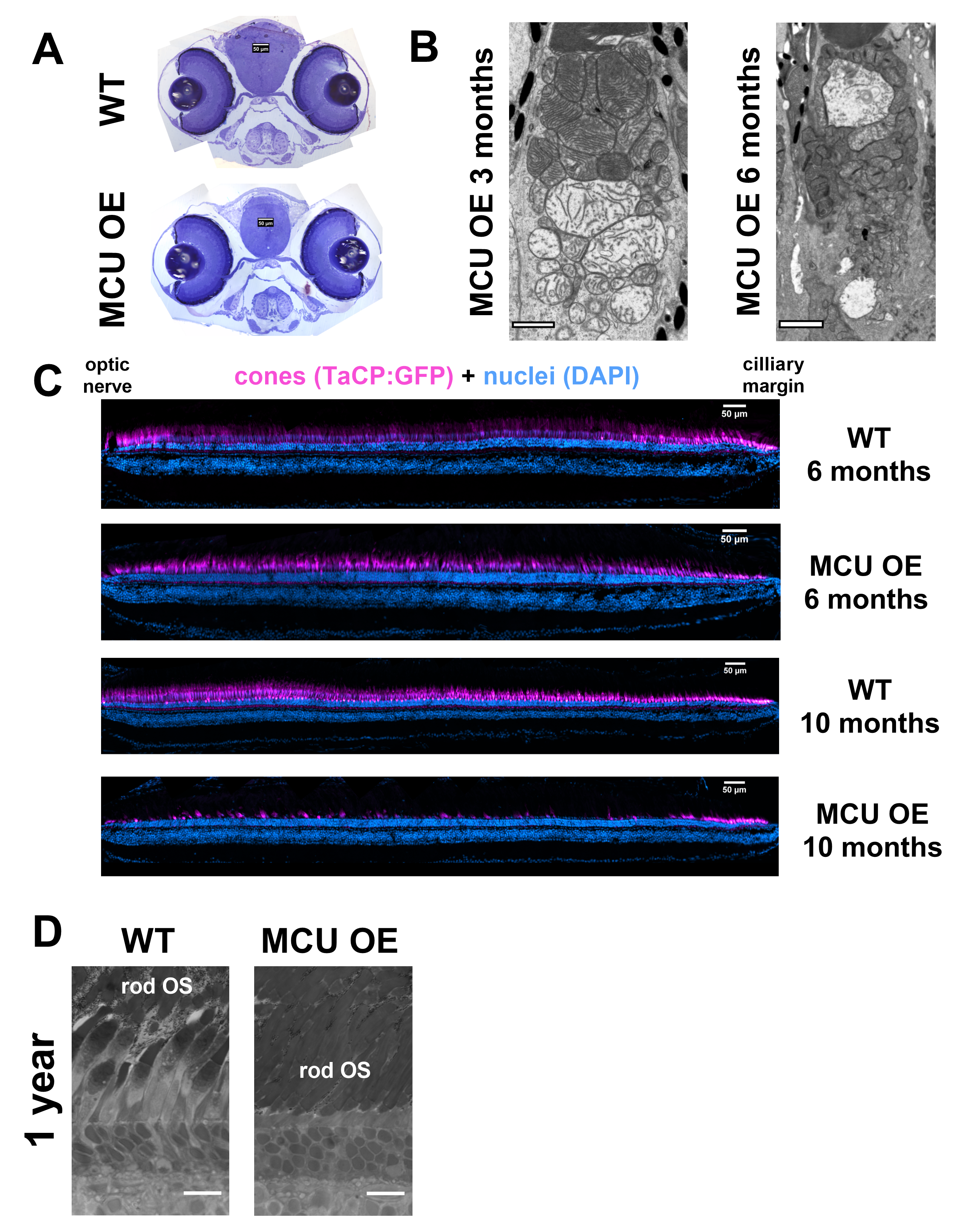

Supplement: Supplementary file 1 — Supplemental Figure 4: Further characterization of retinal health throughout development in both WT and MCU OE models [file 41418_2019_398_MOESM1_ESM.tif]

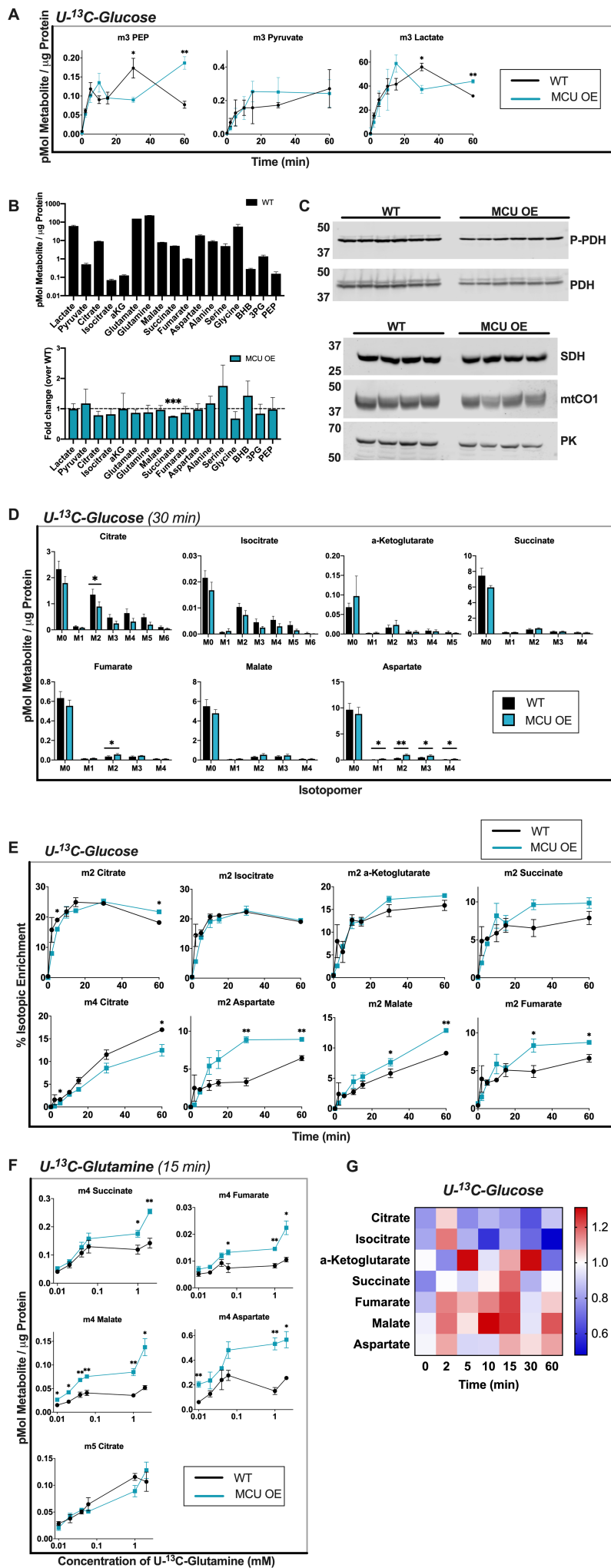

Supplement: Supplementary file 2 — Supplemental Figure 5: Further metabolic characterization of MCU OE retinas [file 41418_2019_398_MOESM2_ESM.pdf]

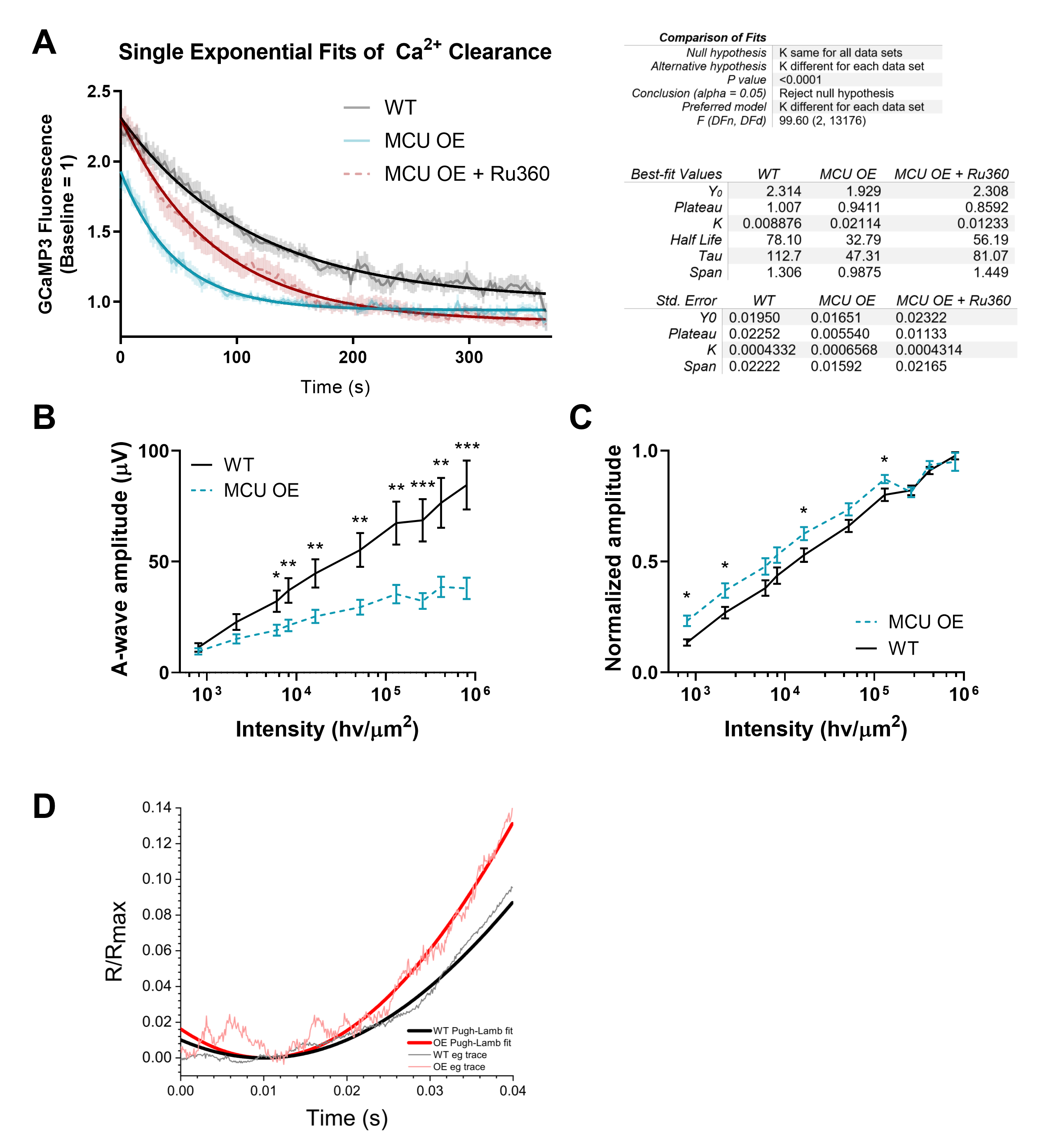

Supplement: Supplementary file 3 — Supplemental Figure 6: Fitting of Ca2+ clearance data and other ERG parameters [file 41418_2019_398_MOESM3_ESM.tif]
