# Supplementary material for: Increasing Ca2+ in photoreceptor mitochondria alters metabolites, accelerates photoresponse recovery, and reveals adaptations to mitochondrial stress
Source: Cell Death Differ. 2019 Aug 2;27(3):1067–85. doi: 10.1038/s41418-019-0398-2 (PMC7206026; doi:10.1038/s41418-019-0398-2)
Supplement: Supplementary file 5 — Supplementary figure legends [file 41418_2019_398_MOESM5_ESM.docx]

**Supplemental Figure Legends**

**Supplemental Figure 4: Further characterization of retinal health throughout development in both WT and MCU OE models.**

A. Larval zebrafish sections at 6 days of age stained with Richardson’s stain. Aside from mitochondrial disturbance in the photoreceptor layer of MCU OE retinas, normal morphology appears to be conserved. Scale bar = 50 µm.

B. EM images of MCU OE fish at 3 months and 6 months of age. Mitochondrial heterogeneity is preserved as MCU OE cones age, with cones containing a mix of both healthy mitochondria and swollen mitochondria. 3 month scale bar = 1 µm, 6 month scale bar = 2 µm.

C. Representative stitched and straightened images of TαCP:GFP (cones, magenta) retinal sections stained with a nuclear stain (Hoescht, cyan) at 6 months and 10 months of age. The double cone nuclei (which sit on top of the nuclear layer that contains both UV/blue cones and rods) were used for quantification. Scale bar = 50 µm.

D. Richardson’s stain of 1 year old zebrafish retina from WT and MCU OE fish. In MCU OE models, cones are rarely observed and instead rods have proliferated (rod OS = rod outer segments). Scale bar = 25 µm.

**Supplemental Figure 5: Further metabolic characterization of MCU OE retinas.**

A. Glycolytic intermediates from 4-month-old WT and MCU OE retinas supplied with ^13^C-glucose. We observed no trends of altered glycolytic flux over time between WT and MCU OE retinas. *p<0.05, **p<0.01 using Welch’s t-test.

B. Total metabolite levels in freshly dissected 4-month-old WT zebrafish retinas and relative levels of these metabolites in MCU OE retinas. (BHB: β-hydroxybutyrate, 3PG: 3-phosphoglycerate, PEP: phosphoenolpyruvate).

C. Top: Immunoblot showing P-PDH and total PDH expression in WT and MCU OE retinas. n=6 WT and 6 MCU OE retinas from 3 different fish. The P-PDH/PDH ratio is 1.12 ± 0.04-fold higher in MCU OE retinas, p<0.05 using Welch’s t-test. Bottom: Immunoblot of MTCO1, SDH, and Pyruvate Kinase (PK) in WT and MCU OE retinas. We observed that expression of every protein we probed for (PDH, SDH, mtCO1 and pyruvate kinase) was slightly lower in MCU OE retinas, even when the same amount of protein lysate was loaded. We hypothesize that this is because in MCU OE retinas, MCU and RFP comprise a much larger fraction of the total protein, so other proteins appear less abundant when normalizing to total protein. n=4 WT and 4 MCU OE retinas from 4 different fish.

D. Distribution of isotopomers in 4-month-old WT and MCU OE retinas fed with U-^13^C-Glucose for 30 min.

E. Isotopic enrichment (µg isotopomer/µg total metabolite) of 4-month-old WT and MCU OE retinas supplied with U-^13^C-Glucose. Since total citrate and isocitrate pools are depleted (see supplemental figure 5G), isotopic enrichment of m2 citrate and m2 isocitrate appear unchanged in MCU OE retinas.

F. Titration of 4-month-old WT and MCU OE retinas supplied with ^13^C-glutamine (0.1, 0.2, 0.4, 0.6, 1, and 2 mM) for 15 minutes. m5 citrate (produced from reductive carboxylation) is included to show that only metabolites directly downstream of α-KGDH are produced at higher levels in MCU OE cones. Data points represent averages from n=3 retinas from 3 different fish. *p<0.05, **p<0.01 using Welch’s t-test.

G. Heat map showing altered distribution of total levels of TCA cycle metabolite in MCU OE retinas fed with U-^13^C-Glucose (relative to WT). At most time points, total levels of citrate and isocitrate are decreased in MCU OE retinas, while total levels of metabolites downstream of α-KGDH are increased.

**Supplemental Figure 6: Fitting of Ca^2+^ clearance data and other ERG parameters.**

A. Truncated Ca^2+^ clearance data used for fitting with a one-phase exponential decay function (least squares fit) in GraphPad Prism 8.0.1. The fitted exponentials are shown by a solid, dark line. No constraints were included for Y_0_ or the plateau, K > 0. K is different for each data set, with p <0.0001. Descriptive statistics of the fit included in table.

B. Absolute amplitude of the isolated a-wave response to varying intensity light of WT and MCU OE retinas for experiments shown in Figure 4D. Bars = standard error. *p<0.05 using Welch’s t-test.

C. Amplitude of a-wave responses in WT and MCU OE retinas normalized to the maximum response for experiments shown in Figure 4D. Bars = standard error. *p<0.05 using Welch’s t-test.

D. Lamb-Pugh model fits to ex-vivo ERG dim flash responses normalized to R_max_ of example WT and OE retinas, with original response traces in pale red/grey. WT mean A = 0.0128 µm^2^s^-2^ ± 0.0143, n= 16 retinas. MCU OE mean A = 0.18278 µm^2^s^-2^ ± 0.0217, n= 23 retinas. *p<0.05 using Welch’s t test.
